# The relationship between faculty interactions, sense of belonging, and academic stress: a comparative study of the post-COVID-19 college life of Korean and international graduate students in South Korea

**DOI:** 10.3389/fpsyt.2023.1169826

**Published:** 2023-05-10

**Authors:** Dongil Kim, Yeyoung Woo, Jusuk Song, Subin Son

**Affiliations:** ^1^Department of Education, Seoul National University, Seoul, South Korea; ^2^Department of Counseling Psychology, Korea Soongsil Cyber University, Seoul, South Korea; ^3^Department of Education, University of Florida, Gainesville, FL, United States

**Keywords:** COVID-19, college life, academic stress, faculty interactions, sense of belonging, graduate students

## Abstract

**Objective:**

Rapid changes in post-COVID-19 higher education have increased students’ academic stress. This study focused on graduate students’ academic stress in South Korea and compared the results for Korean graduate students and those for international graduate students.

**Method:**

Using the online survey results, the study verified the relationships between faculty interactions, a sense of belonging, and academic stress among Korean and international graduate students using a mediating effects analysis and a multigroup path analysis.

**Results:**

The results were as follows. First, Korean students experienced greater academic stress, faculty interactions, and a sense of belonging, but no statistically significant difference was observed. Second, a sense of belonging had a mediating effect on the relationship between faculty interactions and academic stress. Unlike in previous studies, all paths were found to be statistically significant. Faculty interactions had a negative effect on academic stress and a positive effect on a sense of belonging. A sense of belonging had a negative effect on academic stress. Third, the comparison of Korean and international graduate students showed that international students had a greater effect of faculty interactions on academic stress.

**Conclusion:**

Through these results, we explored the post-COVID-19 academic lives of Korean and international graduate students in South Korea and built grounds for effective interventions for alleviating academic stress.

## 1. Introduction

Since March 2020, educational institutions worldwide have experienced rapid changes because of the COVID-19 pandemic. Students began to participate in classes by using online and remote learning tools, which do not require school attendance (1). Higher educational institutions were also affected by the COVID-19 pandemic situation. It changed the traditionally utilized education environment and methods. As online and remote education progressed after COVID-19, college instructors recognized that human encounters had decreased, and negative academic behaviors had increased (2). Students in higher education institutions experienced relief and curiosity as online education began, compared to the education situation before the initial lockdown; however, they also experienced disappointments and perceived certain deficiencies due to the decrease in interactions (3).

Furthermore, this rapid change in higher education environments and practices has increased academic stress (4). For example, college students in China experienced stress because of increased academic burdens and their separation from school after the COVID-19 pandemic (5). Similarly, students in the United States underwent a crisis regarding their academic future, and approximately 30% of students expressed their intention to unregister themselves from their classes or take fewer classes if remote learning and lockdown were to continue (6). These phenomena were similar to those observed in South Korea (henceforth Korea). The stress of college life before and after COVID-19 was qualitatively different as classes and education environments changed fundamentally. Previously, the learning process itself caused academic stress; however, after COVID-19, participating in online learning caused academic stress (7). Students began to feel stressed out because of the lack of differentiation between living spaces and learning spaces, since they were now taking their classes at home. Such academic stress showed a different pattern compared to previous ones in that it was caused by changes in the environment. Preceding studies have confirmed that the academic stress occurring after COVID-19 has been different from previous stress experiences (8); therefore, we must consider ways to deal with this new type of academic stress.

Proper intervention is especially important for dealing with stress because chronic stress negatively affects cognition and causes mental health vulnerabilities (9). As discussed earlier, after the COVID-19 pandemic, students began to experience a new type of academic stress. Moreover, researchers suggest that returning to pre-COVID-19 education is unlikely (10, 11). In other words, non-face-to-face lectures and real-time online lectures may still be maintained, and even without the pandemic, students may still experience different types of academic stress compared to pre-COVID-19. To this end, we verified the variables that could affect academic stress both positively and negatively during the pandemic in higher education institutions. The details on the current research are as follows.

First, the current research was conducted only on graduate students. Graduate students were more susceptible to academic stress during pandemics. For example, students who lived with their children experienced stress because of a decrease in their studying time after the pandemic; furthermore, because of the increase in their economic responsibilities and difficulties, they tended to worry about academic continuation (12). It was also found that graduate students experienced greater stress compared to undergraduate students because of their diversified roles and strengthened responsibilities, even when they were not facing a pandemic situation (13). As such, the Graduate Student Stress Scale is largely divided into academic stress, environmental stress, and stress caused by family and financial responsibilities (14).

Second, among graduate students, the current research focuses on the different experiences between Korean and international graduate students in Korea. The culture of Korean graduate schools tends to exhibit hierarchical and authoritarian relationships (15). Moreover, the teaching style in Korean graduate schools often involves self-directed learning through discussions and presentations (16), and there exists a difference in multicultural sensitivity development between professors with and without experience in studying abroad or supervising international students (17). Consequently, international graduate students who are unfamiliar with Korean culture may experience qualitatively different academic stress, sense of belonging, and faculty interaction compared to Korean graduate students. Especially since the COVID-19, stress and anxiety increased in universities among students who did not move to their homes after the shutdown (18). The Pandemic has made it difficult for international students to return to their home countries, and it is possible that such difficulties have exacerbated these differences. Thus, this study aims to examine experiences related to academic stress after the pandemic among graduate students, while also comparing Korean and international students.

Third, we focused on the role of a sense of belonging and faculty interactions in this regard. Sense of belonging refers to a sense of connection and relationship to people, spaces, places, and so on; it is a concept that includes social place, identity, emotional attachment, and ethical-political values (19). The sense of belonging embodies space and place, and students feel a sense of belonging to the higher education institutions. This sense of belonging felt by university students encompasses judgment on how well they fit into the new school environment, the degree to which they fit in with the values and support promoted within the environment, and the degree to which they integrate themselves with the system (20). Students are influenced by factors such as the memberships they maintain in school and the amount of social support they have (21, 22). Such a sense of belonging can affect their satisfaction, commitment, immersion, and well-being; a low sense of belonging can influence dropout decisions (20, 23). In particular, among graduate students, such sense of belonging has been found to have fundamental differences between PhD students completing their degrees and those who are not (24); furthermore, a sense of belonging to a graduate schools had a positive effect on academic self-concept and commitment to study (25). Thus, in previous studies, a sense of belonging has been found to play the role of a protective factor. Moreover, the strengthened role of a sense of belonging as a protective factor against academic stress after COVID-19 has been confirmed (26).

Faculty interactions affect both students’ sense of belonging and their studies. The instructor variable has a significant effect on students’ learning and motivation, and it also influenced international students’ academic paths. It was confirmed that faculty interactions could affect both academic stress and sense of belonging and that it was an appropriate variable for examining differences between international and domestic students (27). However, there were mixed results from previous studies on the differences between international and domestic students. Kim (28) suggested that in Korea, daily communication with instructors in classes is often conducted in the Korean language, and international students may thus find interactions with instructors more difficult than Korean students because of language barriers. However, in other research, international students perceived higher support from their instructors (29).

**Figure 1:**
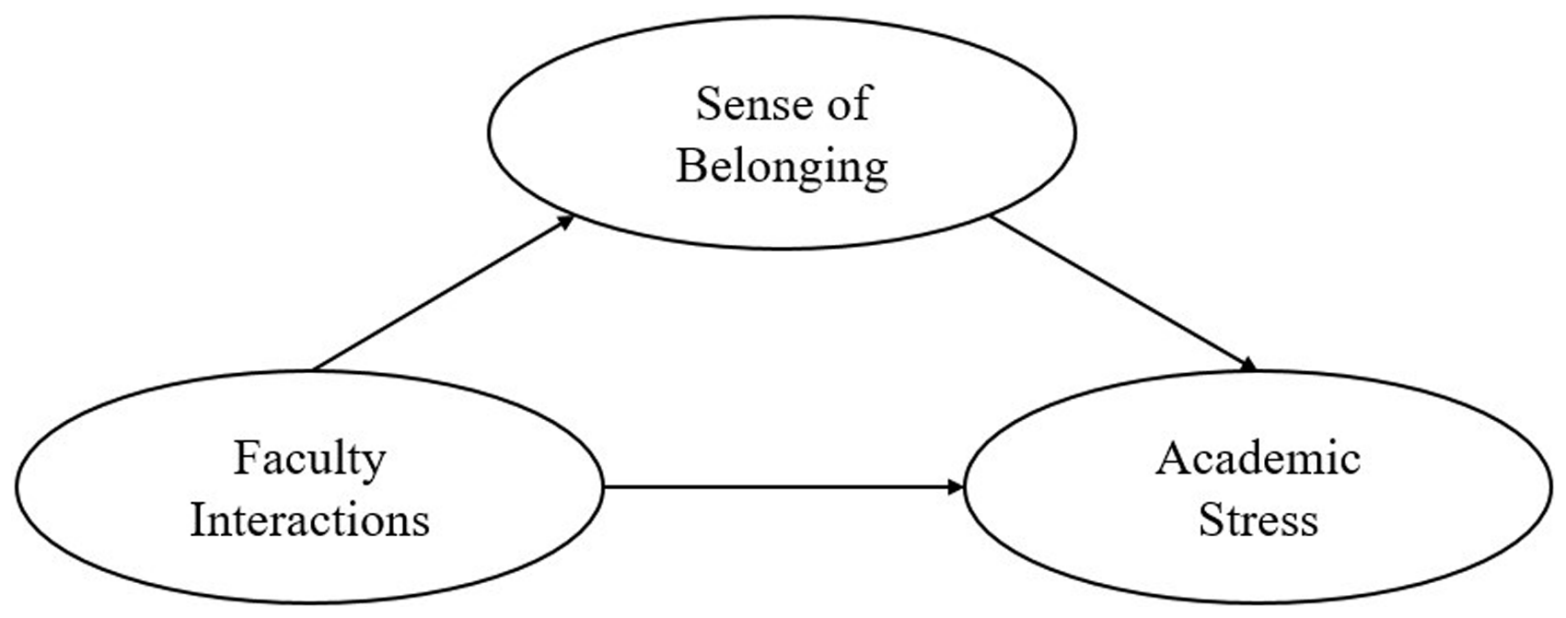
Model figure.

Thus, in this study we focused on the graduate students’ experience on academic stress, sense of belonging, and faculty interactions during the COVID-19 pandemic in Korea, and compared the experience between Korean and international students. By focusing on these variables we expected to broaden the previous studies in new scenes and times because of national differences in Korea and the COVID-19 pandemic. In sum, the relationships between the variables were verified as follows. First, we examined whether there were any differences between the experiences of Korean and international graduate students in terms of academic stress, sense of belonging, and faculty interactions. Furthermore, this current study examined the relationships among the three variables. The research problems and models were as follows (Figure 1):

**RQ1**. Is there a difference in academic stress, sense of belonging, and faculty interactions between Korean and international graduate students?
**RQ2**. Does a sense of belonging mediate the relationship between faculty interaction and academic stress?
**RQ3**. Does the relationship between academic stress, sense of belonging, and faculty interaction differ between Korean graduate students and international graduate students?

## 2. Methods

### 2.1. Participants

The survey participants attended graduate school at S University. The online survey was conducted by the S University Counseling Center for about 1 month (December 21, 2021–January 22, 2022). The counseling center conducted the entire survey utilizing S University’s survey system. In December, the lists of currently enrolled graduate school students were extracted from the survey system, and online questionnaires were delivered to all graduate students via email. Due to the use of secondary data from the S university counseling center, our research was not reviewed by the Institutional Review Board (IRB).

The total number of participants was 2,087. One hundred twenty-three responded data were eliminated from the total participants because of missing values for all items on at least one scale. Regression imputation methods then estimated the remaining missing values using Amos 22.0. The research participants (*n* = 2,087) comprised 1,075 (51.50%) male and 1,012 (48.49%) female participants. The distribution of nationality was as follows: 1,968 (94.30%) Korean students and 119 (5.70%) international students. In terms of degree levels, 1,075 (51.50%) had a master’s degree, 452 (21.66%) had a doctoral degree, and 560 (26.83%) had an integrated Ph.D. Thus, a graduate student referred to the person who has pursued degrees of master, doctoral, and integrated doctoral studies in S university, Korea.

### 2.2. Instruments

#### 2.2.1. Faculty interactions

To measure interactions with the faculty, we used 4 items that were assessed using a 5-point Likert scale (1 = strongly disagree, 5 = strongly agree). Higher item scores indicated that the participants interacted with faculty members more frequently. Each item measured student-faculty interactions regarding academic problems, personal problems, career problems, and the appropriateness of the academic faculty-student ratio. Examples of faculty interaction items were as follows: “Students discuss academic issues (academic plan, fitness of the major, grade, and so on) with faculty members,” “students discuss personal issues in college (economic difficulties, interpersonal problems) with faculty members,” and “My graduate school has an appropriate academic advisor-student ratio.” In this scale, Cronbach’s alpha was 0.83.

#### 2.2.2. A sense of belonging

To measure a sense of belonging, we assessed 9 items using a 5-point Likert scale (1 = strongly disagree, 5 = strongly agree). Higher item scores indicated that the participants perceived a greater sense of belonging. Each item measured subjective perceptions of the available educational opportunities in the relevant college and intimacy with college members. Item 5 was a reversed item. Examples of faculty interaction items were as follows: “I feel my college satisfies my educational goals,” “I feel my college provides me with sufficient opportunities to learn,” and “I feel my college members (cohorts, friends, juniors-seniors) have close enough relationships with me.”In this scale, Cronbach’s alpha was 0.86.

#### 2.2.3. Academic stress

To measure academic stress, we assessed 2 items using a 5-point Likert scale (1 = strongly disagree, 5 = strongly agree). Higher item scores indicated that the participants perceived higher levels of academic stress. Each item measured academic satisfaction and academic problems. Item 1 was a reversed item. Examples of the academic stress items were “the degree of satisfaction with academic and grade management” and “how difficult it has been to deal with academic problems for the past year.” In this scale, Cronbach’s alpha was 0.46 the insufficient level of consistency can be corroborated by the number of the scales. Cronbach’s alpha of 2-item scale can underestimate the true internal consistency (30).

### 2.3. Data analysis

Several steps were taken for conducting data analysis using SPSS 23.0 and Amos 22.0. First, we used descriptive statistics and t-tests to identify the demographic results of the participants, and the mean differences between Korean and international graduate students. Second, a mediating effects analysis was implemented in order to determine the role of a sense of belonging in the relationships between faculty interactions and academic stress. Third, multigroup confirmatory factor analysis was conducted to validate the measurement models for further analysis. Fourth, two multigroup path analyses were conducted to examine the group differences in the structural paths of the tested models.” As the difference between Korean and international graduate students was considered to be the main presenting group difference, our study did not include any confounding factor in subsequent analyses.

## 3. Results

### 3.1. Descriptive statistics

Before conducting the multigroup analysis, the normality of the data and the characteristics of the participants were reviewed. There were 1968 Korean students and 119 international students, and more than half of the students were master students (Korean 51.1%; international 58.8%). The nationalities of the international students (*N* = 119) were as follows: 69 (57.5%) from China, 8 (6.7%) from the United States, 5 (4.2%) from Vietnam, 4 (3.3%) from Taiwan, and 4 (3.3%) from Mongolia.

Before assessing the multigroup path analysis, the normality of the data was reviewed through descriptive statistics. Skewness and kurtosis ranged from −2 to 2, satisfying the assumption of normality. Furthermore, the correlation coefficients did not exceed 0.9 (−0.327 to −0.477), which was acceptable for the multicollinearity test requirements. Finally, a mean difference analysis was performed to compare the scores of each variable based on nationality. There were no significant nationality-based differences in academic stress, faculty interactions, and sense of belonging among graduate students (*p* > 0.05) (see Tables 1–3).

**Table 1:** Descriptive results.

Nationality		*N*	Mean	*SD*	Skewness	Kurtosis
Korean	Academic stress	1968	2.8402	0.73461	0.110	0.264
	Faculty interactions	1968	3.2305	0.91531	−0.077	−0.421
	Sense of belonging	1968	3.6267	0.65237	−0.508	0.521
International	Academic stress	119	2.7941	0.72314	0.154	0.333
	Faculty interactions	119	3.2278	0.98660	0.022	−0.551
	Sense of belonging	119	3.5427	0.59178	0.022	−0.010

SD = Standard deviation.

**Table 2:** Results of path coefficients between structural model variables.

Path	*B*	*S.E*	*β*	*t*
Faculty interactions → Academic stress	−0.121***	0.023	−0.185***	−5.200
Faculty interactions → Sense of belonging	0.698***	0.040	0.548***	17.424
Sense of belonging → Academic stress	−0.260***	0.028	−0.509***	−9.346

S.E = standard error.

***p < 0.001; **p < 0.01; *p < 0.05.

**Table 3:** Indirect effects in structural model.

Path	Indirect effect (β)	Bias-corrected
Faculty interactions → Sense of belonging → Academic stress	−0.279	−0.333 ~ −0.237**

***p < 0.001; **p < 0.01; *p < 0.05.

### 3.2. Mediating effect

The structural equation model fit was examined in order to identify the structural models related to a sense of belonging, faculty interactions, and academic stress, and the model fit was as follows: *χ*^2^ = 3336.207 (df = 87), CFI = 0.814, NFI = 0.810, IFI = 0.813, and RMSEA = 0.134. Though the study reported poor fit of RMSEA, the model fit could be marginally accepted due to the other fit values such as CFI ≥ 0.8 and IFI ≥ 0.8 (31, 32) The path coefficients were statistically significant (*p* < 0.001) (Table 2). Faculty interactions had a negative effect on academic stress (*β* = −0.185) and a positive effect on a sense of belonging (*β* = 0.548). A sense of belonging had a negative effect on academic stress (*β* = −0.509). The indirect effect of a sense of belonging was statistically significant (*β =* −0.279, *p* < 0.01) (Table 3).

### 3.3. Multigroup confirmatory factor analysis (multigroup CFA)

Multigroup CFA was conducted between the data of Korean and international graduate students in order to assess measurement invariance. First, an unconstrained model (*χ*^2^ = 3406.996 (*p* < 0.001), TLI = 0.743, CFI = 0.814, IFI = 0.815, RMSEA = 0.094) demonstrated an acceptable model fit as CFI, IFI, and RMSEA scores could be acceptable even if these are at the marginal. Thus the study proceeded to the measurement invariance for further steps. Following that, the difference tests comparing between the unconstrained and constrained models revealed that constrained models 1 (factor loadings invariance model) and 2 (covariance invariance model) were not significantly different at the significance levels of 0.05 (*p* > 0.05). The multigroup model was satisfied with configural measurement, factor loadings, and covariance invariance models, which allowed for multigroup path analyses. However, the constrained 3 (factor loadings and covariance invariance model) and 4 (factor loadings and Covariance and error invariance model) were significantly different from each other (*p* < 0.001).

### 3.4. Multigroup path analysis

All paths between faculty interactions, academic stress, and sense of belonging were statistically significant, but group differences were only significant in the path between faculty interactions and academic stress (Table 4). In the Korean student group, interactions with faculty negatively impacted academic stress (*β* = −0.167, *p* < 0.001). Furthermore, the faculty interactions of international students also had a negatively significant effect on academic stress (*β* = −0.556, *p* < 0.01); however, the data of international students had a greater standardized coefficient value than that of Korean students. Therefore, significant differences were noted between the groups in the path of faculty interactions and academic stress (*p* < 0.05).

**Table 4:** Results of multigroup path analysis.

Path	Korean students			International students			Diff
	*B*	*β*	*S.E*	*B*	*β*	*S.E*	
Faculty interactions → Sense of belonging	0.707^***^	0.545^***^	0.042	0.611^***^	0.580^***^	0.127	−0.714
Faculty interactions → Academic stress	−0.108^***^	−0.167^***^	0.023	−0.388^**^	−0.556^**^	0.129	−2.134^*^
Sense of belonging → Academic stress	−0.265^***^	−0.512^***^	0.029	−0.225^*^	−0.338^*^	0.106	0.283

S.E = standard error.

## 4. Discussion

First, a comparison of the experiences of international graduate students and Korean graduate students revealed that the latter had more academic stress, faculty interactions, and a sense of belonging; nonetheless, no statistically significant difference between both student groups was found. This result differed from that of previous studies. Except for a sense of belonging the present study reported contrasting outcomes in terms of academic stress and faculty interactions (29). The interpretations of the results were as follows. Academic stress may stem from the strong passion and emphasis on higher education in Korea. According to a study conducted in Korea, the academic stress scores of college students were in the high-risk stress levels within the top 30% of the normative range (33). Considering previous research indicating that graduate students experience higher levels of academic stress compared to undergraduate students (12, 34), it can be expected that graduate students in Korea also experience significantly high levels of academic stress. In short, overly high expectations and enthusiasm for higher education in Korea might have led to higher scores of academic stress for Korean students than those for international students.

Furthermore, in terms of faculty interactions, Korean laboratory’s hierarchical culture and the composition of instructors may have influenced the results (15). In a previous study on school adaption of international graduate students, “scary advisor” and “rules in laboratory community life that they have not experienced in their home countries” were identified as social stressors (35). In other words, international students may have perceived their professors as more unapproachable due to the distinctive culture of Korean graduate school laboratories; thus, such cultural differences may have led them to decrease interactions with their faculties. Korean graduate students have also suggested that a neglectful culture and unfriendly and insincere education are parts of the features of Korean graduate schools (36). Also, differences in faculty interactions may have resulted from the experience and composition of the instructors. A study on Korean instructors’ multicultural sensitivity toward international graduate students discovered that instructors’ experiences influenced their teaching of international graduate students (17). Instructors who have never studied abroad or taught international students are still in the early stages of development. Based on these prior studies in Korea, it can be predicted that the current study’s findings would differ from those of previous studies in the United States due to the different atmosphere and instructors at Korean graduate schools.

Second, unlike previous studies, all paths were found to be significant in the relationship between academic stress, faculty interactions, and a sense of belonging experienced by international students. The relationship between a sense of belonging and academic stress was found to be generally consistent with previous studies. Although no prior studies have directly revealed the relationship between sense of belonging and academic stress experienced by international graduate students, previous studies have revealed that college student’s sense of belonging is negatively related to stress (37), and positively related to academic motivation, academic endurance and persistence, and academic value (38). Levett-Jones and Lathlean (39) stated that the unfulfilled desire to belong can increase academic stress or anxiety, making it hard for international students to fully focus on their studies or exert their academic competence. In particular, international students in college need psychological stability because they not only face stress as a result of other students’ negative perceptions on their special admissions (40) but also their sense of belonging (or lack thereof) (41). According to Chen and Zhou (42), cultural influence, information accessibility, language barriers, and various other factors can influence international students’ academic performance, causing academic stress to rise during the adaptation process. Therefore, in a challenging academic environment, it is critical for students to have the experience of being accepted and having their psychological needs met by others around them; this can reduce their academic stress.

The prior research showed findings of both directions between the relationship between a sense of belonging and faculty interactions (43, 44). Kim et al. (44) proved that a sense of belonging can significantly affect faculty interactions because a higher sense of belonging could lead to development of positive feelings and prosocial behaviors. On the other hand, faculty interactions can also influence students’ sense of belonging (43). Faculty interactions are essential human resources for educational purposes; the level of proactive interactions is considered a key aspect in maintaining academic relationships (45). In other words, frequent faculty interactions in a graduate setting can promote a greater sense of belonging. Graduate students tend to identify current positions throughout their graduate programs; hence, faculty interactions can play a positive role in students’ adaptation process. Furthermore, with the growing interest in informal interactions such as personal interactions and counseling (46), as well as academic interactions such as assignments, exams, and grades, various and active interactions with professors (45) are helpful for international graduate students to experience enough sense of belonging and reduce their academic stress in a graduate setting.

Third, significant differential effects of faculty interactions on academic stress have been revealed between Korean and international graduate students. Both groups reported that faculty interactions had a negative effect on academic stress, but this effect was significantly greater among international students. This finding is consistent with previous studies (27, 47–50). Rice et al. (50) concluded that a relationship with the instructor is a significant factor to relieve academic stress among international students. Although moderating effects between Korean and international students were not revealed, there is convincing evidence that international students are more vulnerable to a lack of respects from their instructors compared to Korean students (50). Furthermore, the role of the instructor has been proven as the most influential factor for international students to decide whether they continue their academic studies (27). Similarly, the Chinese international students in Korea, satisfaction with their interactions with professors reduced stress in adaptation to classes [Cho and Jeon, 2009; (49)] or increased participation in the class (47). Therefore, as a university-level policy, appropriate plans or programs should be considered as ways of strengthening interactions between instructors and students to alleviate the academic stress among international graduate students. Increased faculty interactions can contribute to international students’ successful acculturation and academic achievement.

The significance and further directions of this study are as follows. First, only a handful of previous studies have examined the academic stress experienced by Korean graduate students. Most prior research looked at the stress issue with narrow scope to medical graduate students (51). Further research must be conducted to explore diverse majors or general graduate students encompassing their academic stress issues. Second, this current research is essential for finding ways to deal with their mental health considering the higher stress levels of graduate students than undergraduates. In particular, the COVID-19 pandemic situation shed light on the importance of examining the stress issues of Korean graduate students. This is because the pandemic situation has posed serious mental health issues and required timely and appropriate mental health interventions (52). Thus, current study can be a primary source to suggest and organize useful interventions for aiding mental health and college adaptation interventions. The limitations of this study are as follows. First, the external validity issue might be threatened because participants were collected only in S University graduate students in Seoul, Korea. Thus, the study findings may not be fully representative of graduate students. Second, there was a considerable gap in the ratio of Korean to foreign students. The number of recruited international students in this study was smaller than the actual percentage of international students in the University. Therefore, future research should prepare appropriate measures to increase international graduate participants as well as diverse universities to generalize research findings to the population.

## Data availability statement

The original contributions presented in the study are included in the article/supplementary material, further inquiries can be directed to the corresponding author.

## Author contributions

DK had the idea for the article. YW, JS, and SS performed the literature search and data analysis. YW and DK drafted the main idea for the first draft, and wrote it with JS and SS. YW revised the work, and DK supervised the whole process of the research from the data collection. All authors contributed to the article and approved the submitted version.

## Funding

This work was supported by the National Research Foundation of Korea grant funded by the Korean Government (NRF-2020S1A3A2A02103411).

## Conflict of interest

The authors declare that the research was conducted in the absence of any commercial or financial relationships that could be construed as a potential conflict of interest.

## Publisher’s note

All claims expressed in this article are solely those of the authors and do not necessarily represent those of their affiliated organizations, or those of the publisher, the editors and the reviewers. Any product that may be evaluated in this article, or claim that may be made by its manufacturer, is not guaranteed or endorsed by the publisher.

## References

[1] UNESCO COVID-19 Impact on Education. Available at: https://en.unesco.org/covid19/educationresponse (accessed March 13, 2021) (2020).

[2] Moralista R Oducado RM. Faculty perception toward online education in a state college in the Philippines during the coronavirus disease 19 (COVID-19) pandemic. Univ J Educ Res. (2020) 8:4736–42. doi: 10.13189/ujer.2020.081044

[3] Karalis T Raikou N. Teaching at the times of COVID-19: inferences and implications for higher education pedagogy. Int J Acad Res Bus Soc Sci. (2020) 10:479–3. doi: 10.6007/IJARBSS/v10-i5/7219

[4] Moawad RA. Online learning during the COVID-19 pandemic and academic stress in university students. Revista Românească pentru Educaţie Multidimensională. (2020) 12:100–7. doi: 10.18662/rrem/12.1sup2/252

[5] Yang C Chen A Chen Y. College students’ stress and health in the COVID-19 pandemic: the role of academic workload, separation from school, and fears of contagion. PloS One. (2021) 16:e0246676. doi: 10.1371/journal.pone.0246676, PMID: 33566824 PMC7875391

[6] Clabaugh A Duque JF Fields LJ. Academic stress and emotional well-being in United States college students following onset of the COVID-19 pandemic. Front Psychol. (2021) 12:628787. doi: 10.3389/fpsyg.2021.628787, PMID: 33815214 PMC8010317

[7] Moon HJ. A phenomenological study of stress experience of college students displayed in non-face-to-face college life by COVID-19. J Learner-Centered Curric Instruct. (2021) 21:233–47. doi: 10.22251/jlcci.2021.21.11.233

[8] Ibda H Wulandari TS Abdillah A Hastuti AP Mahsum M. Student academic stress during the COVID-19 pandemic: a systematic literature review. Int J Public Health Sci. (2023) 12:286–5. doi: 10.11591/ijphs.v12i1.21983

[9] Marin MF Lord C Andrews J Juster RP Sindi S Arsenault-Lapierre G. Chronic stress, cognitive functioning and mental health. Neurobiol Learn Mem. (2011) 96:583–95. doi: 10.1016/j.nlm.2011.02.016 21376129

[10] Horváth D Ásványi K Cosovan A Csordás T Faludi J Galla D. Online only: future outlooks of post-pandemic education based on student experiences of the virtual university. Soc Econ. (2022) 44:2–21. doi: 10.1556/204.2021.00026

[11] O'Dea XC Stern J. Virtually the same?: online higher education in the post Covid-19 era. Br J Educ Technol. (2022) 53:437. doi: 10.1111/bjet.13211 35600417 PMC9111677

[12] Nodine PM Arbet J Jenkins PA Rosenthal L Carrington S Purcell SK. Graduate nursing student stressors during the COVID-19 pandemic. J Prof Nurs. (2021) 37:721–8. doi: 10.1016/j.profnurs.2021.04.008, PMID: 34187670 PMC8245865

[13] Johnson B Batia AS Haun J. Perceived stress among graduate students: roles, responsibilities, & social support. Vahperd J. (2008) 29:31–6.

[14] Rocha-Singh IA. Perceived stress among graduate students: development and validation of the graduate stress inventory. Educ Psychol Meas. (1994) 54:714–7. doi: 10.1177/0013164494054003018

[15] Jon JE Jeon H Lee H. Graduate advisors’ advising types and graduate students’ intent to drop out: analysis by the field of study. Korean Educ Rev. (2021) 27:407–3. doi: 10.29318/KER.27.1.15

[16] Kwon Y. The sociocultural adjustment of Chinese graduate students at Korean universities: a qualitative study. Int J Intercult Relat. (2013) 37:536–9. doi: 10.1016/j.ijintrel.2013.06.004

[17] Kim Y Kim G Jeon Y. The study on the developing process in multicultural sensitivity of foreign graduate Students' Korean advisor. Stud Human. (2013) 37:461–8.

[18] Husky MM Kovess-Masfety V Swendsen JD. Stress and anxiety among university students in France during Covid-19 mandatory confinement. Compr Psychiatry. (2020) 102:152191. doi: 10.1016/j.comppsych.2020.152191, PMID: 32688023 PMC7354849

[19] Cuervo H Wyn JA. longitudinal analysis of belonging: temporal, performative and relational practices by young people in rural australia. Young. (2017) 25:219–34.

[20] Hoffman M Richmond J Morrow J Salomone K. Investigating “sense of belonging” in first-year college students. J. Coll. Stud. Retent. Res. Theory Pract. (2002) 4:227–56.

[21] Hagerty BM Lynch-Sauer J Patusky KL Bouwsema M Collier P. Sense of belonging: A vital mental health concept. Arch. Psychiatr. Nurs. (1992) 6:172–77. 1622293 10.1016/0883-9417(92)90028-h

[22] Hagerty BM Williams RA Coyne JC Early MR. Sense of belonging and indicators of social and psychological functioning. Arch. Psychiatr. Nurs. (1996) 10:235–44. 8799050 10.1016/s0883-9417(96)80029-x

[23] Gopalan M Brady ST. College students’ sense of belonging: A national perspective. Educ. Res. (2020) 49:134–7. 10.3102/0013189x19897622 PMC11156233 38847009

[24] Lovitts BE. Leaving the ivory tower: The causes and consequences of departure from doctoral study. Lanham: Rowman & Littlefield Publishers (2002).

[25] Ostrove JM Stewart AJ Curtin NL. Social class and belonging: implications for graduate students' career aspirations. J High Educ. (2011) 82:748–74.

[26] Procentese F Capone V Caso D Donizzetti AR Gatti F. Academic community in the face of emergency situations: sense of responsible togetherness and sense of belonging as protective factors against academic stress during covid-19 outbreak. Sustainability. (2020) 12:9718. doi: 10.3390/su12229718

[27] Glass CR Kociolek E Wongtrirat R Lynch RJ Cong S. Uneven experiences: the impact of student-faculty interactions on international Students' sense of belonging. J Int Stud. (2015) 5:353–7. doi: 10.32674/jis.v5i4.400

[28] Kim J. International students’ intercultural sensitivity in their academic socialisation to a non-English-speaking higher education: a Korean case study. J Furth High Educ. (2020) 44:939–5. doi: 10.1080/0309877X.2019.1627298

[29] Curtin N Stewart AJ Ostrove JM. Fostering academic self-concept: advisor support and sense of belonging among international and domestic graduate students. Am Educ Res J. (2013) 50:108–7. doi: 10.3102/0002831212446662

[30] Eisinga R Grotenhuis M Pelzer B. The reliability of a two-item scale: Pearson, Cronbach, or spearman-Brown? Int J Public Health. (2013) 58:637–2. doi: 10.1007/s00038-012-0416-3 23089674

[31] Akkuş A. Developing a scale to measure students’ attitudes toward science. Int J Assess Tools Educ. (2019) 6:706. doi: 10.21449/ijate.548516

[32] Kim H Ku B Kim JY Park YJ Park YB. Confirmatory and exploratory factor analysis for validating the phlegm pattern questionnaire for healthy subjects. Evid Based Complement Alternat Med. (2016) 2016:2696019. doi: 10.1155/2016/2696019 27051447 PMC4804052

[33] Cha NH. The relationships between academic stress and adjustment at university life in Korean university students. J Korean Acad Community Health Nurs. (2016) 27:124–1. doi: 10.12799/jkachn.2016.27.2.124

[34] Lee SI Cho CH Lee YY Lee JH Shin JM Jung DY. Mediating effects of depression and anxiety on the relationship between stress and suicidal tendency of Institute of Science and Technology students: focusing on the comparison between degree courses. J Rehab Psychol. (2019) 26:1–18.

[35] Park M Kim Y Hong E. A study of international graduate students’ adaptation to school life in Korea-focusing on stress and adaptation process. Teach Korean Foreign Lang. (2014) 40:109–07.

[36] Lim HJ Kim SH Park HY Kim K. A study on master students‘graduate school experience in a Korean research university. Asian J Educ. (2016) 17:379–8. doi: 10.15753/aje.2016.09.17.3.379

[37] Grobecker PA. A sense of belonging and perceived stress among baccalaureate nursing students in clinical placements. Nurse Educ Today. (2016) 36:178–3. doi: 10.1016/j.nedt.2015.09.015, PMID: 26471423

[38] Goodenow C Graddy K. The relationship of school belonging and friend’s values to academic motivation among urban adolescent students. J Exp Educ. (1993) 62:60–71. doi: 10.1080/00220973.1993.9943831

[39] Levett-Jones T Lathlean J. The ascent to competence conceptual framework: an outcome of a study of belongingness. J Clin Nurs. (2009) 18:2870–9. doi: 10.1111/j.1365-2702.2008.02593.x, PMID: 19220619

[40] Nho CR. A study on university adjustment among returnees of overseas experienced students-focusing on relationships among stress, perception of university environment, life satisfaction, and self-esteem, and service needs. Mental Health Social Work. (2002) 13:87.

[41] Glass CR Westmont CM. Comparative effects of belongingness on the academic success and cross-cultural interactions of domestic and international students. Int J Intercult Relat. (2014) 38:106–9. doi: 10.1016/j.ijintrel.2013.04.004

[42] Chen J Zhou G. Chinese international students’ sense of belonging in north American postsecondary institutions: a critical literature review. Brock Educ J. (2019) 28:48–63. doi: 10.26522/brocked.v28i2.642

[43] Choi B. Exploring influences of student characteristics and early college experiences on the change rate of the sense of belonging of Korean local undergraduate students. Korea Educ Rev. (2021) 27:289–4. doi: 10.29318/KER.27.3.11

[44] Kim J Jung S Kim E. The mediating effects of professor-student interactions between the sense of school belonging and student engagement of university students. J Think Dev. (2019) 15:1–29. doi: 10.51636/JOTD.2019.12.15.3.1

[45] Park SM Park JH. An analysis of the effectiveness of the interaction promotion program with Faculty for Freshmen: K university case. J Learn Center Curric Instruct. (2020) 20:1045–69. doi: 10.22251/jlcci.2020.20.18.1045

[46] Astin AW. What matters in college? Four critical years revisited. San Francisco: Jossey-Bass (1993).

[47] Bae S Hong J. The effects of student-faculty interaction and supportive campus climate on Chinese students’ active classroom engagement. Korean J Comp Educ. (2013) 23:109–4.

[48] Cho H Jon K. Factors associated with living satisfaction of studying in Korean universities. J Soc Sci. (2009) 20:193–3.

[49] Lee H. Factors affecting depression of the Chinese students in Korea: focusing on socioeconomic characteristics, language skills, acculturative stress and social support. J Korean Soc Well. (2012) 7:129–3.

[50] Rice KG Suh H Yang X Choe E Davis DE. The advising alliance for international and domestic graduate students: measurement invariance and implications for academic stress. J Couns Psychol. (2016) 63:331–2. doi: 10.1037/cou0000141, PMID: 27045448

[51] Lee WS Oh YJ Byun DY. The academic stress, depression and social support of graduate medical school students: testing the buffering effect of social support. Kor J Soc Welfare Res. (2013) 37:45–70.

[52] Moreno C Wykes T Galderisi S Nordentoft M Crossley N Jones N. How mental health care should change as a consequence of the COVID-19 pandemic. Lancet Psychiatry. (2020) 7:813–24. doi: 10.1016/S2215-0366(20)30307-2, PMID: 32682460 PMC7365642

